# Elevated levels of preβ1-high-density lipoprotein are associated with cholesterol ester transfer protein, the presence and severity of coronary artery disease

**DOI:** 10.1186/s12944-016-0394-1

**Published:** 2017-01-10

**Authors:** Xiao-min Bu, Dong-mei Niu, Jia Wu, Yun-long Yuan, Jia-xi Song, Jun-jun Wang

**Affiliations:** Department of Clinical Laboratory, Jinling Hospital, School of Medicine, Nanjing University, 305East Zhongshan Rd., Nanjing, 210002 China

**Keywords:** Preβ1-HDL, CETP, Coronary artery disease, Atherosclerosis

## Abstract

**Background:**

Preβ1-high-density lipoprotein (preβ1-HDL), plays an important role in reverse cholesterol transport and exhibits potent risk for coronary artery disease (CAD). However, the association of plasma preβ1-HDL and cholesterol ester transfer protein (CETP) levels in CAD patients and the relationship of preβ1-HDL with extent of CAD are debatable.

**Methods:**

Preβ1-HDL and CETP levels were measured by enzymed-linked immunosorbent assay (ELISAs) in 88 acute coronary syndromes (ACS), 79 stable coronary artery disease (SCAD) patients and 85 control subjects. The correlation analyses, multiple linear regression analyses and logistic regression analyses were performed, respectively.

**Results:**

The preβ1-HDL and CETP levels in ACS patients were significantly higher than those in SCAD patients and both of them were higher than controls’. Preβ1-HDL levels were positively associated with CETP (*R* = 0.348, *P* = 0.000), the diameter of stenosis (*R* = 0.253, *P* = 0.005), the number of vessel disease (*R* = 0.274, *P* = 0.002) and Gensini score (*R* = 0.227, *P* = 0.009) in CAD patients. Stepwise multiple linear regression analyses showed that CETP was one of the determinants of preβ1-HDL levels. Logistic regression analysis revealed that elevated preβ1-HDL and CETP were potential risk factors for both ACS and SCAD.

**Conclusion:**

The elevated preβ1-HDL levels may change with CETP concentrations in CAD patients and were related to the presence and severity of CAD.

## Background

Preβ1-high-density lipoprotein (preβ1-HDL), a quantitatively minor high-density lipoprotein (HDL), is formed as nascent apolipoprotein (apo) A-I enters plasma and as a substrate or product in interconversion of HDL species [1]. After receiving free cholesterol effluxed from ATP binding cassette transporter A1 (ABCA1) transporter, it is esterified with a free fatty acid derived from lecithin mediated by lecithin-cholesterol acyl transferase (LCAT) [2, 3]. Cholesteryl esters (CE) are then incorporated into cores of α-HDL, subsuming preβ1-HDL. CE are transferred from α-high-density lipoprotein into cores of accepter lipoproteins by cholesterol ester transfer protein (CETP) [4]. The preβ1-HDL was shown to be significantly correlated to the high efflux which was mediated by ABCA1 [5]. The fasting preβ1-HDL concentration has been reported elevated in patients with coronary artery disease (CAD), hyperlipidemia, type 2 diabetic, obesity and hemodialysis [6–9]. It is proposed that increased preβ1-HDL may be used as a marker of risk for structural CAD, myocardial infarction (MI) and cerebral vascular disease (CVD) [10]. However, a recent study reported that serum preβ1-HDL levels detected by their new one-dimensional polyacrylamide gel electrophoresis (PAGE) system were negatively associated with severity of CAD [11]. This result needs further validition. Actually, the relationship between preβ1-HDL and CAD is still controversial.

CETP, one of decisive factors that determines high-density lipoprotein cholesterol (HDL-C), mediates the transfer of CE from HDL to low density lipoprotein (LDL), very low density lipoprotein (VLDL), and an exchange of triglyceride (TG). It relates to particle size, lipid composition and function of lipoprotein [12]. CETP also plays a key role in reverse cholesterol transport (RCT) and development of atherosclerosis [13, 14]. Several lines of evidence have shown that incubation of large α-HDL particles with CETP could produce preβ1-HDL [6, 7]. which suggested the levels of preβ1-HDL may be associated with CETP. An earlier research performed in transgenic mice indicated that the expression of CETP resulted in an increase in the proportion of apoA-I in the preβ1-HDL fraction and a stimulation of the efflux and esterification of cell-derived cholesterol [15]. However, study has been published to show the relationship of plasma preβ1-HDL and CETP levels in CAD patients. Therefore, this study was undertaken to investigate the associations of plasma preβ1-HDL and CETP in CAD patients, and to further elucidate the clinical values of preβ1-HDL for evaluating the severity of CAD.

## Methods

### Study subjects

A total of 167 admitted CAD patients were randomly enrolled from the Department of Cardiology of Jinling Hospital between January 2014 and March 2015. All the patients were undergoing clinically indicated coronary angiography. Angiograms of all the CAD patients showed at least 50% stenosis in ≥1 coronary artery. 88 patients with acute coronary syndromes (ACS) included acute myocardial infarction patients and unstable angina (UA) with Braunwald classification II or III, who exhibited the positive cardiac biomarkers result [cardiactroponin I (cTnI) > 0.090 ng/ml], acute ischemic-type chest pain (lasting for > 15 min, duration from symptoms onset to emergency admission within 72 h) and characteristic electrocardiogram changes. 79 stable coronary artery disease (SCAD) patients with a normal electrocardiogram and documented normal left ventricular contractility, except for possible minor nonspecific ST-T features, had a minimum 1-year history without any cardiac events/procedures suggestive of ACS. The exclusion criteria of the CAD patients included mild disease of angiography (a stenosis of 10 to 50% of the luminal diameter in all the 3 coronary arteries), prior coronary revascularization, and the presence of renal disease. 85 control subjects selected from routine health examination were found normal in physical and electrocardiography and laboratory tests, and without diseases such as hyperlipidemia, hypertension, diabetes mellitus, or any clinical evident sign of atherosclerosis. In patients with ACS, blood samples were taken on admission. Blood samples were collected at least 12 h after fasting from control subjects and patients with SCAD. The blood sample was collected into EDTA (1 mg/ml) containing tube and plasma was promptly separated by a 15 min centrifugation at 3000 rpm, then stored at −80 °C until analysis. This study protocol was approved by the Ethics Committee of Jinling Hospital (REC number: GH23335H) and all the subjects provided written informed consent.

### Angiographic analysis

Catheterization was performed by either the Sones or the Judkins. Multiple views including angulated views were obtained, and the angiograms were evaluated. The extent of angiographically documented CAD was quantified in the left anterior descending coronary artery, the left circumflex artery, or the right coronary artery as follows: normal coronary arteries (smooth, with either no stenosis or a stenosis of <10% of the luminal diameter), mild disease (a stenosis of 10 to 50% of the luminal diameter in one or more coronary arteries), or one-vessel, two-vessel, or three-vessel disease, defined as a stenosis of more than 50% of the luminal diameter in one, two, or three coronary arteries. To eliminate bias of judgment, angiographic observation and laboratory assays were conducted by different investigators in a double-blind way until all results were recorded and ready for statistical analysis. The severity of CAD was determined according to the Gensini score based on the degree of luminal stenosis and its geographical importance [16]. Reductions in the luminal diameter of 1–25%, 26–50%, 51–75%, 76–90%, 91–99% and total occlusion were respectively evaluated as 1, 2, 4, 8, 16 and 32. Each principal vascular segment was multiplied by a factor in accordance with the functional importance of the myocardial area supplied by that segment, that is, the left main (LM) was assigned a multiplier × 5; the proximal segment of the left anterior descending (LAD) and the proximal segment of the left circumflex (LCX) × 2.5; the middle of the LAD × 1.5; the distal segment of the LAD, the right coronary artery (RCA), the posterolateral artery, and the obtuse marginal artery × 1; and other small vascular branches × 0.5 [17].

### Assays

Preβ1-HDL in human plasma was measured by a commercial preβ1-HDL sandwich enzymed-linked immunosorbent assay (ELISA) kits from Sekisui Medical Co. LTD as described [18]. CETP was measured by sandwich ELISA as our laboratory described [19, 20]. TC, TG, high density lipoprotein (HDL) cholesterol were measured (Daiichi Pure Chemicals, Japan) on a Hitachi 7600 analyzer. Low-density lipoprotein cholesterol (LDL-C) was estimated using the Friedewald formula [21]. Creatine kinase-MB (CK-MB) was studied with Roche diagnostic kits in a COBAS C-8000 Roche autoanalyzer and studied by an immunological UV method, and cardiac troponin I (cTnI) tests were studied with TOSOH ST AIA-PACK cTnI 3-rd-Gen kit by AIA-2000ST TOSOH autoanalyzer.

### Statistic analysis

Statistic analyses were performed with SPSS 16.0 and the values were expressed as the mean ± standard deviation (SD). One Sample Kolmogorov-Smirnov Test was used to evaluate the normality of distribution of the variables. The skewed data, such as preβ1-HDL, CETP and TG concentrations were log-transformed to create a more normal distribution. The differences of variants among groups were analyzed by one-way analysis of variance (ANOVA), and the differences between groups were subsequently determined by Fisher LSD test when appropriate. The difference in the extent of angiographically documented disease, cTnI and CK-MB between two groups was analyzed by Student’s *t*-test. The differences in sex among groups and medication were analyzed by the Chi-square test. Correlations between preβ1-HDL, CETP, Gensini score, cTnI and other variables in CAD patients were calculated by the non-parametric Spearman rank coefficient test. The stepwise multiple linear regression analyses (*P*_in_ = 0.05, *P*_out_ = 0.10) were used to identify the influencing factor for plasma preβ1-HDL levels. The univariate and multivariate logistic regression analyses were used to calculate the approximation of the relative risk, odds ratio (OR) and 95% confidence interval (CI) for selected variables. A two-tailed *P*-value less than 0.05 was considered statistically significant.

## Results

### Baseline clinical characteristics in the study group

The baseline clinical characteristics of the patients and control subjects, indications for coronary disease, and lipid measurements are showed in Table 1. There was no significant difference in age or sex among the ACS or SCAD patients and control subjects (*P* > 0.05). 54.5% of ACS and 31.6% of SCAD patients received statin treatment in the present study at the time of sampling. The extent of angiographically documented CAD in the ACS patients was greater than that in SCAD patients (Table 1).

**Table 1:** Baseline clinical characteristic, lipid concentrations in the study groups

Variable	ACS (*n* = 88)	SCAD (*n* = 79)	Control (*n* = 85)
Age (y)	64.52 ± 12.04	67.42 ± 13.34	63.79 ± 13.17
Male/female	62/26	56/23	64/21
Hypertension (%)	59 (67%)	62 (78%)	0 (0)
Ischemic stroke (%)	32 (36%)	24 (30%)	0 (0)
Diabetes mellitus (%)	39 (44%)	30 (38%)	0 (0)
Medication			
Statins, n (%)	48 (54.5)	25 (31.6)	0 (0)
ACEI/ARB, n (%)	63 (71.6)	51 (64.6)	0 (0)
Beta blockers, n (%)	58 (65.9)	42 (53.2)	0 (0)
Aspirin (%)	60 (68.2)	26.2 (21.0)	0 (0)
Angiographic analysis			
Maximal stenosis (%)	89.34 ± 15.62^**^	23.87	-
Number of vessel disease	2.04 ± 0.85	1.14 ± 0.93	-
cTnI, ng/L	19.42 ± 71.19^**^	0.03 ± 0.02	-
CK-MB, U/L	75.42 ± 126.14^**^	11.68 ± 4.12	
Gensini score	62.23 ± 41.58^**^	17.20 ± 18.39	-

Data are presented as the mean ± SD or number (%) of subjects

Compared with SCAD: ^*^ *P* < 0.05, ^**^ *P* < 0.01

### Plasma preβ1-HDL, CETP and lipid concentrations in ACS and SCAD patients

Compared with control subjects, preβ1-HDL and CETP levels were found significantly increased in both patients with ACS and SCAD. Furthermore, preβ1-HDL and CETP levels were significantly higher in ACS than those in SCAD (Table 2).

**Table 2:** Plasma preβ1-HDL, CETP and other lipid concentrations in ACS, SCAD and control groups

Variable	ACS (*n* = 88)	SCAD (*n* = 79)	Control (*n* = 85)
TC(mmol/L)	4.68 ± 1.34	4.16 ± 1.10	4.62 ± 0.58
TG(mmol/L)	2.04 ± 1.47^**^	1.70 ± 1.31^**^	1.09 ± 0.41
HDL-C(mmol/L)	0.99 ± 0.23^**^	1.13 ± 0.72^**^	1.31 ± 0.25
LDL-C(mmol/L)	2.91 ± 1.02^*^	2.88 ± 0.85^*^	2.45 ± 0.81
Preβ1-HDL(μg/L)	36.24 ± 17.03^**,#^	24.68 ± 21.02^**^	7.44 ± 5.49
CETP (mg/L)	3.42 ± 1.80^**,##^	2.05 ± 1.10^*^	1.52 ± 0.98

Compared with control: ^*^ *P <* 0.05; ^**^ *P <* 0.01

Compared with SCAD: ^#^ *P <* 0.05; ^##^ *P <* 0.01

### Associations among preβ1-HDL, CETP, other lipid levels and the extent of CAD

To study the relationship of preβ1-HDL, CETP, other lipid parameters and the extent of CAD, Spearman rank correlation analyses were performed. In all the CAD patients, including ACS and SCAD patients, preβ1-HDL were found positively correlated with CETP (*r* = 0.348, *P* = 0.000), maximal stenosis (*r* = 0.253, *P* = 0.005), number of vessel disease (*r* = 0.274, *P* = 0.002), Gensini score (*r* = 0.227, *P* = 0.009), TC (*r* = 0.401, *P* = 0.000), TG (*r* = 0.195, *P* = 0.017) and LDL-C (*r* = 0.309, *P* = 0.000); and CETP were positively correlated with number of vessel disease (*r* = 0.238, *P* = 0.013), Gensini score (*r* = 0.282, *P* = 0.002), TC (*r* = 0.209, *P* = 0.016) and LDL-C (*r* = 0.202, *P* = 0.023) (Table 3).

**Table 3:** Spearman rank correlations between preβ1-HDL, CETP and other variable in CAD patients

Variable	Preβ1-HDL	CETP
TC	0.401^**^(*P* = 0.000)	0.209^*^(*P* = 0.016)
TG	0.195^*^(*P* = 0.017)	0.126 (*P* = 0.150)
HDL-C	0.052 (*P* = 0.537)	−0.0335 (*P* = 0.692)
LDL-C	0.309^**^ (*P* = 0.000)	0.202^*^(*P* = 0.023)
CETP	0.348^**^(*P* = 0.000)	-
Preβ1-HDL	-	0.348^**^(*P* = 0.000)
Maximal stenosis(%)	0.253^**^ (*P* = 0.005)	0.087(*P* = 0.370)
Number of vessel disease	0.274^**^ (*P* = 0.002)	0.238^*^(*P* = 0.013)
Gensini score	0.227^**^ (*P* = 0.009)	0.282^**^ (*P* = 0.002)

^*^ *P <* 0.05; ^**^ *P <* 0.01

To further explore the possible factors affecting preβ1-HDL levels in CAD patients, the multiple linear regression analyses was performed. Consequently, the TC, CETP accounted for 19.9% of the variation of preβ1-HDL levels, when all variables that were significantly correlated with preβ1-HDLwere included as independent variables (Table 4).

**Table 4:** Stepwise multiple linear regression analyses of factors affecting preβ1-HDL levels in CAD patients

	Unstandardized coefficients		Standardized coefficient	*P* value
	B	SE	Beta	
Independent variables in the model				
Constant				
TC	8.448	1.917	0.371	0.000
CETP^a^	2.706	1.216	0.187	0.028
Independent variables excluded from model				
TG^a^			0.086	0.354
LDL-C^a^			−0.045	0.743

The dependent variable was preβ1-HDL (adjusted R^2^ = 0.199). TC, TG, LDL-C, Ox-LDL and CETP were used for independent variables

^a^These variables were log-transformed before analyses

### Preβ1-HDL and CETP as risk factors for ACS and SCAD

The univariate and multivariate logistic regression analyses were next performed to evaluate the possible associations of preβ1-HDL and CETP with CAD. As shown in Table 5, using the control group as the reference category when the ACS, SCAD or control group was treated as a dependent three-category variable, the univariate analyses revealed that increased preβ1-HDL or CETP was the potential risk factor for ACS (preβ1-HDL, OR = 1.161, 95% CI: 1.107–1.219, *P* < 0.001; CETP, OR = 3.055, 95% CI: 2.158–4.326, *P* < 0.001), and for SCAD (preβ1-HDL, OR1.142, 95% CI: 1.089–1.198, *P* < 0.001; CETP, OR = 1.632, 95% CI: 1.188–2.217, *P* < 0.05). To further evaluate the associations of preβ1-HDL and CETP with the severity of CAD in ACS or SCAD patients, preβ1-HDL (OR = 1.017, 95% CI: 1.003–1.031, *P* = 0.016) and CETP (OR = 1.931, 95%CI: 1.456–2.563, *P* < 0.001) were found closely associated with ACS group when treated the SCAD group as the reference category.

**Table 5:** Logistic regression analysis of risk factors for ACS and SCAD

	Preβ1-HDL		CETP	
	OR(95%CI)	*P* value	OR(95%CI)	*P* value
Univariate analyses^a,b^				
Model^1^				
ACS	1.161 (1.107–1.219)	<0.001	3.055 (2.158–4.326)	<0.001
SCAD	1.142 (1.089–1.198)	<0.001	1.623 (1.188–2.217)	0.002
Model^2^				
ACS	1.017 (1.003–1.031)	0.016	1.931 (1.456–2.563)	<0.001
Multivariate analyses^a,c^				
Model^1^				
ACS	1.236 (1.118–1.366)	<0.001	3.077 (1.765–5.364)	<0.001
SCAD	1.207 (1.094–1.332)	<0.001	1.866 (1.105–3.152)	0.02
Model^2^				
ACS	1.024 (1.002–1.046)	0.031	1.682 (1.221–2.318)	0.001

Model^1^ : the reference category was the control group

Model^2^ : the reference category was the SCAD group

^a^In univariate and multivariate logistic regression analyses, the ACS, SCAD or control group was treated as a dependent three-category variable and the OR was considered statistically significant when the lower limit of the 95% CI was > 1.0 and the *P*-value was < 0.05

^b^Only one of Preβ1-HDL, CETP was included in the univariate analyses

^c^The age, gender and coexisting conditions were adjusted in the multivariate analyses

After adjusting for age, gender and plasma lipid levels and using the control group as the reference category, when the ACS, SCAD or control group was treated as a dependent three-category variable, the increased preβ1-HDL or CETP was also revealed to be the potential risk factor for both ACS (preβ1-HDL, OR =1.236, 95% CI: 1.118–1.366, *P* < 0.001; CETP, OR = 3.077, 95% CI: 1.765–5.364, *P* < 0.001) and SCAD (preβ1-HDL, OR = 1.207, 95% CI: 1.094–1.332, *P* < 0.001; CETP, OR = 1.866, 95% CI: 1.105–3.152, *P* = 0.020). Similarly, preβ1-HDL or CETP was also showed closely association with ACS group when treated the SCAD group as the reference category.

## Discussion

This study for the first time shows that the elevated preβ1-HDL levels were related to the presence and severity of CAD and changed with CETP levels in CAD patients. Compared with control subjects, the plasma levels of preβ1-HDL and CETP were significantly increased in both SCAD and ACS patients. CETP was found to be one of potential predictors for preβ1-HDL levels. Furthermore, the preβ1-HDL levels in ACS patients were significantly higher than those in SCAD patients, and were significant risk for both ACS and SCAD patients.

Preβ1-HDL has been reported to be increased in CAD patients [10]. Amar et al. found that preβ1-HDL concentrations were 2-fold higher in individuals with ischemic heart disease (IHD) than no IHD groups [22]. Besides, it has been reported that preβ1-HDL level was markedly higher in the unstable angina pectoris (UAP) subgroup than in the stable angina pectoris (SAP) subgroup [23, 24]. In this study, we found that the preβ1-HDL levels increased in CAD patients and it’s levels in ACS patients were significantly higher than those in SCAD patients, which was consistent with earlier observations [23, 24] suggesting that the increased preβ1-HDL concentrations might be one of the major factors for the occurrence and severity of CAD. In addition, we further found that plasma preβ1-HDL was positively related with degree of coronary artery stenosis, number of vessel disease and Gensini score, suggesting that the increased preβ1-HDL may associated with cholesterol accumulation in coronary artery vessels. Miyazaki et al. [25] and Sethi et al. [22] also found that the preβ1-HDL level was high in hyperlipidemic and ischemic heart disease subjects. In our and the above studies, preβ1-HDL level was measured by ELISA using anti-human preβ1-HDL monoclonal antibody (MAb 55201) specifically reacted with apoA-I associated with preβ1-HDL in plasma. Recently, Chen et al. described that serum preβ1-HDL levels were decreased and negatively associated with Gensini score in patients with coronary artery diseases [11], which was inconsistent with the above and previous studies [10, 23, 24]. Chen et al. isolated the preβ1-HDL using a native polyacrylamide gel electrophoresis (PAGE) system with polyclonal anti-human apoA-I and detected the levels of preβ1-HDL by densitometry, which is a semi-quantitative method. Whereas, ELISA is a quantitative method. Although significantly high preβ1-HDL levels were observed in CAD cases, the association between preβ1-HDL level and CAD prevalence was inconsistent. Bela et al. reported that the increased preβ1-HDL was not significantly associated with CAD prevalence but associated with recurrent CAD events [26, 27]. A large well-characterized clinical cohort study confirmed the association between increased levels of preβ1-HDL and risk of CAD, and proposed that high levels of preβ1-HDL remained a significant predictor of CAD even after adjustment for traditional risk factors [10].

Preβ1-HDL is considered to be an atherogenic lipoprotein; although Lynnda et al. found that preβ-HDL formation was affected by high-normal free thyroxine in type 2 diabetes mellitus [28]. The mechanisms by which it contributes to atherosclerosis remain widely unknown. It is important to realize that the RCT pathway is a cyclical way [10, 29, 30]. The increase of preβ1-HDL may be indicative of a defect in one part of the pathway. It has been reported that the causes of increased preβ1-HDL included the defect of ABCA1 transporter, low levels of LCAT, overexpression of phospholipid transfer protein (PLTP) or increased plasma cholesterol efflux capacity from macrophages [3, 31–33]. Increasing evidence suggested that the concentration and composition of plasma lipids and lipoproteins were markedly affected by the acute phase reactions, which associated with injury, inflammation or sepsis [34]. The changes of the concentration and composition of plasma HDL in patients with ACS could be secondary to systemic acute phase response. As an acute phase protein mainly produced in the liver, levels of serum amyloid A (SAA) both in plasma and HDL compositions increase markedly in inflammation [34]. It has been suggested that these changes in HDL apoprotein pattern could increase HDL catabolism, impair reverse cholesterol transport. This would reverse the flux of cholesterol between macrophages and HDL and result in increased macrophage uptake. Therefore, the changes of the composition of plasma HDL in patients with ACS that secondary to systemic acute phase response were proinflammatory. Preβ1-HDL, as a minor HDL, may be secondary to systemic acute phase response in ACS [35]. Future research should focus on the role of preβ1-HDL in this process.

Several lines of evidence suggested that preβ1-HDL concentration associated with CETP level [36]. When plasma was incubated with exogenous CETP at 37 °C, total preβ1-HDL concentration increased [37]. In cholesterol-fed rabbits, serum preβ1-HDL concentration and CETP activity were 32 and 33% higher than those in control rabbits respectively [38]. And the plasma preβ1-HDL concentration was positively correlated with the hepatic CETP mRNA level [38]. In a human study, probucol increased CETP mass by 20%, and maintained a high preβ1-HDL concentration in hypercholesterolemics [39]. However, Nicholls et al. found that evacetrapib, potent CETP inhibitor significantly increased preβ1-HDL levels in dyslipidemic patients [40]. Our present results further confirmed that the plasma preβ1-HDL levels elevated with high CETP mass in patients with CAD. As we all known, CETP plays an important role in the transfer and exchange of CE and TG among lipoproteins, so that it affects lipid composition, concentration, particle size and function of lipoprotein [12]. In addition, it is now accepted that virtually all of the efflux mediated by the ABCA1 transporter goes to preβ1-HDL, which contains only one molecular species of protein, apoA-I. Then their unesterified cholesterol is esterified by the action of LCAT, and subsumed into larger cholesteryl ester-rich HDL particles from which the esters can be transferred to acceptor lipoproteins, including VLDL and LDL, mediated by CETP. Thus, the present results indicated that in CAD patients, with the increasing CETP concentration, more preβ1-HDL were regenerated during the CETP-mediated transfer process. The current study supports the concept that incubation of large α-HDL particles with CETP may produce preβ1-HDL, and increased preβ1-HDL may be an indicator in the reverse cholesterol transport pathway, which may be closely related to the presence of CAD.

The limitations of this study include the fact that the control subjects were not examined by coronary angiography to exclude potential CAD. This is mainly limited by the wishes of the individual volunteers; however, we have performed physical, electrocardiography and laboratory tests for the control subjects and make sure that all of the controls are without diseases such as hyperlipidemia, hypertension, diabetes mellitus, or any clinical sign of atherosclerosis. In addition, subjects using statin were not excluded from the present study, fortunately, statin treatment decreases preβ1-HDL so that it can reveal better the increase of preβ1-HDL in CAD [40, 41]. Besides, future studies should directly capture and analyze the embolized debris for preβ1-HDL and CETP.

## Conclusions

The present study demonstrated that elevated plasma concentrations of preβ1-HDL reflect the presence and extent of angiographically documented CAD, especially clinically expressed in ACS. The plasma preβ1-HDL levels were potently correlated with CETP in CAD, and may change with CETP concentrations. These finding will contribute to the understanding of the pathogenetic role of preβ1-HDL and CETP in CAD. Further studies are needed to validate these associations and to elucidate pathophysiologic mechanisms of plasma preβ1-HDL in CAD.

## Abbreviations

ABCA1: ATP binding cassette transporter A1
ACS: Acute coronary syndromes
ANOVA: One-way analysis of variance
apo: Apolipoprotein
CAD: Coronary artery disease
CE: Cholesteryl ester
CETP: Cholesterol ester transfer protein
CI: Confidence interval
CK-MB: Creatine kinase-MB
cTnI: Cardiac troponin I
CVD: Cerebral vascular disease
ELISA: Enzymed-linked immunosorbent assay
HDL: High-density lipoprotein
HDL-C: High-density lipoprotein cholesterol
IHD: Ischemic heart disease
LAD: Left anterior descending
LCAT: Lecithin-cholesterol acyl transferase
LCX: Left circumflex
LDL: Low-density lipoprotein
LDL-C: Low-density lipoprotein cholesterol
LM: The left main
MI: Myocardial infarction
OR: Odds ratio
PAGE: Polyacrylamide gel electrophoresis
PLTP: Phospholipid transfer protein
preβ1-HDL: Preβ1-high-density lipoprotein
RCT: Reverse cholesterol transport
SAP: Stable angina pectoris
SCAD: Stable coronary artery disease
SD: Standard deviation
TC: Total cholesterol
TG: Triglyceride
UAP: Unstable angina pectoris
VLDL: Very low density lipoprotein
